# Supplementary material for: A Likelihood Approach for Real-Time Calibration of Stochastic Compartmental Epidemic Models
Source: PLoS Comput Biol. 2017 Jan 17;13(1):e1005257. doi: 10.1371/journal.pcbi.1005257 (PMC5240920; doi:10.1371/journal.pcbi.1005257)
Supplement: S1 File — (TAR.GZ) [file pcbi.1005257.s014.tar.gz › HSPH_Online-SI-Revision/IPoi09/n10-severe/n10-severe_BENCH_table-8.pdf]

1	R0	{1.68952, 11.8402}	{1.88812, 11.4266}	{{1.62191, 8.12461}, {2.16755, 14.8261}}	{14.6924, 19.9428}	inf	217.	512.232	{130., 898.}	153.135	0.395685	newly	155.	365.75	{88., 702.}	154.516	0.400233	Reff	1.60166	1.71802	{1.27904, 2.18367}	16.0892	ar	6872.	7731.59	{6481., 8507.}	14.4819
2	R0	{1.71739, 9.62661}	{1.26246, 3.30679}	{{1.25474, 3.26445}, {1.26656, 3.29313}}	{26.4899, 65.6495}	inf	89.	115.962	{11., 211.}	63.1374	0.307347	newly	86.	277.115	{20., 501.}	240.564	0.538951	Reff	1.66844	1.16747	{1.04796, 1.25047}	30.0263	ar	6874.	3817.42	{3391., 4271.}	44.4658
3	R0	{1.48936, 6.23975}	{1.36, 3.46992}	{{1.24751, 2.5364}, {1.50982, 4.79544}}	{14.5059, 50.7667}	inf	75.	149.906	{30., 340.}	141.375	0.427723	newly	102.	318.781	{83., 661.}	227.114	0.442385	Reff	1.45406	1.37056	{1.04465, 1.9672}	19.5992	ar	5666.	5102.66	{3330., 8060.}	25.7462
4	R0	{1.62394, 7.51135}	{1.89087, 16.1375}	{{1.66328, 11.8799}, {2.11476, 19.5406}}	{17.151, 114.841}	inf	51.	150.642	{26., 308.}	214.391	0.516222	newly	60.	76.4717	{16., 149.}	75.0629	0.371561	Reff	1.59877	1.84929	{1.58471, 2.00689}	16.4217	ar	6417.	7500.83	{6796., 8283.}	20.7659
5	R0	{1.53632, 8.57652}	{1.25546, 4.29133}	{{1.21159, 3.57392}, {1.38815, 6.71734}}	{18.8149, 51.2457}	inf	91.	160.84	{74., 240.}	91.956	0.363323	newly	100.	262.76	{97., 375.}	171.4	0.510138	Reff	1.48962	1.06799	{0.938261, 1.27932}	28.304	ar	6260.	3559.72	{2894., 5102.}	43.1355
6	R0	{1.47166, 5.7434}	{1.92349, 16.1638}	{{1.67375, 12.272}, {2.12588, 19.5406}}	{30.8303, 181.433}	inf	45.	107.608	{22., 237.}	162.832	0.383588	newly	63.	53.4902	{14., 111.}	59.5394	0.363554	Reff	1.45297	1.93425	{1.72743, 2.15726}	33.1242	ar	5650.	7727.51	{7240., 8423.}	40.6722
7	R0	{1.48179, 8.87369}	{1.9897, 12.4303}	{{1.80156, 10.0119}, {2.22867, 15.6411}}	{34.2768, 40.6849}	inf	250.	584.95	{108., 1062.}	157.06	0.398257	newly	229.	394.433	{70., 733.}	100.655	0.328537	Reff	1.37169	1.71751	{1.34673, 2.04172}	27.4598	ar	5233.	7836.23	{7143., 8486.}	49.7465
8	R0	{1.53669, 6.47807}	{2.34729, 14.1962}	{{1.87476, 9.64595}, {2.85956, 18.4275}}	{52.7497, 119.143}	inf	77.	168.484	{21., 396.}	157.163	0.413878	newly	107.	116.063	{14., 247.}	78.4463	0.411758	Reff	1.50611	2.32791	{1.8072, 2.79375}	54.5643	ar	5934.	8631.75	{7709., 9313.}	45.4626
9	R0	{1.57227, 6.20989}	{1.76161, 5.35197}	{{1.19264, 2.14605}, {2.52681, 9.79002}}	{27.7435, 44.3456}	inf	9.	44.3404	{2., 103.}	422.931	0.756265	newly	13.	89.1915	{2., 222.}	612.93	0.841024	Reff	1.56598	2.02315	{1.3258, 2.86463}	36.023	ar	6136.	6938.81	{4454., 9361.}	36.8845
10	R0	{1.54936, 6.50938}	{2.15878, 6.58123}	{{1.4031, 2.81014}, {2.82466, 9.89059}}	{42.2835, 27.9745}	inf	30.	65.5645	{6., 165.}	158.118	0.49823	newly	40.	135.806	{9., 356.}	270.726	0.57954	Reff	1.53789	2.22853	{1.64442, 2.8058}	46.8566	ar	6275.	7894.32	{5694., 9276.}	37.8491
11	R0	{1.42179, 3.18601}	{1.31743, 2.39361}	{{1.19264, 2.01465}, {1.37363, 2.81014}}	{7.34014, 24.8712}	inf	3.	126.292	{4., 276.}	4120.83	1.42328	newly	12.	482.167	{17., 975.}	3933.33	1.47997	Reff	1.40601	1.22293	{1.11073, 1.33489}	13.0212	ar	5322.	3889.75	{2680., 4927.}	26.9119
12	R0	{1.48035, 6.46183}	{2.32532, 11.0733}	{{1.59034, 5.4396}, {2.7943, 15.1048}}	{57.7604, 77.3156}	inf	26.	51.4925	{5., 112.}	135.247	0.467223	newly	35.	62.0746	{4., 159.}	127.079	0.465539	Reff	1.47147	2.32823	{1.66361, 2.89969}	59.3277	ar	5793.	8193.39	{6270., 9335.}	49.0675
13	R0	{1.75755, 8.53854}	{2.00101, 14.8557}	{{1.59682, 9.52864}, {2.39393, 19.3849}}	{18.6268, 77.0002}	inf	33.	91.2258	{16., 211.}	203.421	0.485816	newly	37.	51.8226	{6., 114.}	99.3461	0.446765	Reff	1.74472	2.03646	{1.68619, 2.33818}	18.2813	ar	6832.	7649.95	{6881., 8767.}	21.7442
14	R0	{1.47355, 4.89451}	{2.0673, 10.1159}	{{1.28596, 3.47086}, {2.76203, 15.6124}}	{44.4889, 115.713}	inf	20.	69.5333	{7., 145.}	278.667	0.515104	newly	45.	70.2833	{5., 138.}	117.074	0.459564	Reff	1.4625	2.16132	{1.44019, 2.82264}	49.1225	ar	5710.	7796.95	{5393., 9274.}	45.2572
15	R0	{1.62071, 5.98665}	{2.20416, 4.10319}	{{1.31023, 2.14605}, {2.9097, 5.76688}}	{42.8991, 35.3482}	inf	11.	9.46	{0., 27.}	110.545	1.1282	newly	20.	70.61	{0., 157.}	358.15	1.36589	Reff	1.61763	2.20018	{1.3101, 2.90825}	42.8578	ar	6635.	4679.03	{1., 9260.}	61.5185
16	R0	{1.67268, 7.7256}	{1.37879, 3.41899}	{{1.24751, 2.5364}, {1.5451, 4.64686}}	{17.9578, 55.7446}	inf	103.	198.941	{10., 392.}	131.354	0.539392	newly	121.	466.	{23., 839.}	309.869	0.754685	Reff	1.63555	1.26224	{1.03617, 1.49557}	23.0956	ar	6947.	4552.06	{2861., 6277.}	35.118
17	R0	{1.67805, 11.1417}	{2.00177, 8.44775}	{{1.31535, 3.47086}, {2.81617, 14.003}}	{28.8985, 36.3564}	inf	109.	213.818	{43., 405.}	132.694	0.394979	newly	87.	258.255	{31., 675.}	221.087	0.473898	Reff	1.646	2.04958	{1.2801, 2.77836}	34.3808	ar	6625.	7786.33	{5174., 9334.}	27.3863
18	R0	{1.66743, 9.99046}	{1.60006, 6.21728}	{{1.31535, 3.47086}, {2.01028, 10.1103}}	{17.8575, 46.3053}	inf	118.	182.487	{25., 453.}	105.932	0.420639	newly	114.	243.026	{24., 451.}	152.901	0.46456	Reff	1.61574	1.65812	{1.26524, 2.46128}	21.426	ar	6899.	6545.77	{4767., 9007.}	21.1112
19	R0	{1.70787, 9.85478}	{1.29484, 4.50452}	{{1.25656, 3.82585}, {1.34002, 5.27199}}	{24.1837, 54.2911}	inf	143.	218.5	{60., 388.}	79.9428	0.290031	newly	120.	348.727	{124., 538.}	198.939	0.447111	Reff	1.63409	1.09587	{0.926839, 1.29057}	32.9369	ar	6867.	4349.41	{3913., 4818.}	36.6622
20	R0	{1.53809, 10.5848}	{1.22493, 3.76254}	{{1.21159, 3.57392}, {1.24719, 4.06287}}	{20.3601, 64.4533}	inf	127.	157.52	{111., 225.}	34.4252	0.131538	newly	110.	288.88	{210., 375.}	162.618	0.405022	Reff	1.48364	1.01156	{0.900721, 1.12359}	31.8188	ar	6108.	3465.16	{3047., 3787.}	43.2685
21	R0	{1.59925, 6.74479}	{2.43733, 7.80206}	{{1.79887, 4.61852}, {2.9194, 10.3743}}	{53.0605, 27.1626}	inf	35.	72.7015	{13., 119.}	134.158	0.357053	newly	45.	126.015	{26., 235.}	196.617	0.421204	Reff	1.58662	2.45939	{1.95276, 2.89634}	55.763	ar	6411.	8718.85	{7881., 9346.}	37.3439
22	R0	{1.38779, 4.40977}	{1.44736, 3.66603}	{{1.36035, 3.22945}, {1.47608, 4.06661}}	{6.39884, 19.6081}	inf	43.	171.735	{12., 349.}	330.301	0.633015	newly	75.	408.147	{27., 802.}	469.922	0.746931	Reff	1.36101	1.38149	{1.21438, 1.46988}	7.89696	ar	5154.	5268.09	{4440., 5917.}	16.7323
23	R0	{1.53657, 7.50352}	{1.78341, 7.58078}	{{1.31535, 3.47086}, {2.58655, 14.3523}}	{24.3742, 44.5962}	inf	100.	247.702	{27., 549.}	178.298	0.459285	newly	116.	302.106	{27., 779.}	188.023	0.453074	Reff	1.50353	1.80756	{1.27197, 2.72391}	30.5034	ar	5928.	7094.96	{4845., 9241.}	28.2543
24	R0	{1.71131, 6.24526}	{1.32001, 4.13602}	{{1.09005, 2.14605}, {1.47651, 5.4396}}	{27.1211, 48.8913}	inf	12.	42.1333	{5., 88.}	279.444	0.554873	newly	18.	95.2333	{3., 210.}	456.481	0.686563	Reff	1.70104	1.35384	{1.064, 1.85541}	25.7705	ar	6924.	3864.17	{132., 7447.}	47.9246
25	R0	{1.4412, 3.07729}	{2.17951, 5.78038}	{{1.37363, 2.55684}, {2.82914, 8.62388}}	{53.325, 94.4117}	inf	5.	30.1639	{1., 57.}	530.164	0.742004	newly	20.	76.918	{5., 141.}	321.148	0.643877	Reff	1.43789	2.32593	{1.64772, 2.9419}	63.1882	ar	5810.	7475.85	{21., 9350.}	53.466
26	R0	{1.4159, 4.93858}	{2.46235, 9.02959}	{{1.83544, 5.70177}, {2.93597, 11.6547}}	{74.1482, 86.233}	inf	18.	43.4853	{6., 86.}	168.056	0.431546	newly	30.	62.6176	{8., 131.}	144.216	0.425633	Reff	1.40925	2.50203	{1.88187, 2.9268}	77.5438	ar	5255.	8634.38	{7506., 9376.}	67.2474
27	R0	{1.57465, 7.05414}	{2.05805, 12.0217}	{{1.29927, 6.0993}, {2.7943, 17.2462}}	{38.04, 78.9202}	inf	15.	4.9726	{0., 13.}	69.4064	0.834153	newly	22.	4.35616	{0., 13.}	81.8182	1.19034	Reff	1.57134	2.0859	{1.33948, 2.80513}	39.6905	ar	6359.	5929.32	{1., 9260.}	48.9292
28	R0	{1.41422, 4.62402}	{2.18887, 10.8985}	{{1.28596, 4.21286}, {2.86501, 15.7026}}	{58.139, 142.093}	inf	6.	18.4167	{1., 45.}	248.611	0.565187	newly	14.	18.65	{0., 43.}	114.405	0.646852	Reff	1.41153	2.32784	{1.77435, 2.87859}	65.4109	ar	5485.	7566.78	{8., 9319.}	59.1483
29	R0	{1.41901, 3.63017}	{1.91498, 11.8662}	{{1.23086, 5.61163}, {2.70836, 18.727}}	{41.3591, 227.078}	inf	3.	1.82	{0., 4.}	73.3333	0.667445	newly	4.	1.81	{0., 4.}	83.25	0.971541	Reff	1.4183	1.91441	{1.23074, 2.70809}	41.3769	ar	5979.	3415.5	{1., 9028.}	72.9791
30	R0	{1.60719, 4.72984}	{1.95419, 11.8373}	{{1.14639, 6.01426}, {2.688, 17.5607}}	{33.4584, 151.684}	inf	1.	2.12	{0., 5.}	174.	0.522516	newly	2.	2.45	{0., 6.}	137.5	0.808934	Reff	1.60687	1.95354	{1.14628, 2.68719}	33.4405	ar	6479.	3818.58	{1., 8989.}	66.9643
31	R0	{1.38175, 6.26468}	{1.37609, 2.58162}	{{1.27121, 1.91565}, {1.4804, 3.12918}}	{8.23617, 61.742}	inf	64.	129.613	{33., 253.}	128.125	0.426941	newly	96.	474.548	{82., 913.}	407.359	0.70771	Reff	1.35577	1.40598	{1.16481, 1.50953}	15.6384	ar	5397.	4963.45	{3947., 6118.}	24.58
32	R0	{1.4947, 8.8847}	{1.42658, 5.32128}	{{1.42659, 5.32185}, {1.42659, 5.32185}}	{4.55742, 40.1074}	inf	208.	402.852	{198., 533.}	99.1631	0.284686	newly	186.	519.037	{289., 710.}	180.167	0.424377	Reff	1.38021	1.02967	{0.773964, 1.3156}	25.3974	ar	5407.	5303.19	{5014., 5547.}	3.19545
33	R0	{1.55891, 8.28978}	{2.18949, 16.8363}	{{1.86094, 12.6353}, {2.42625, 19.5287}}	{40.4501, 103.097}	inf	149.	519.074	{135., 1114.}	256.102	0.471585	newly	142.	258.093	{75., 521.}	102.334	0.289814	Reff	1.49203	1.96855	{1.69144, 2.23855}	32.0435	ar	6239.	8372.3	{7597., 8840.}	34.1929
34	R0	{1.60764, 10.4729}	{1.59373, 7.76768}	{{1.44485, 6.00596}, {1.81647, 10.5184}}	{9.21553, 28.3522}	inf	256.	501.8	{164., 813.}	111.738	0.332689	newly	188.	507.525	{145., 721.}	178.684	0.436831	Reff	1.49125	1.28195	{1.00027, 1.66788}	19.2201	ar	6495.	6213.	{5289., 7452.}	12.1901
35	R0	{1.48527, 5.74485}	{1.96592, 8.26892}	{{1.30094, 3.26445}, {2.73634, 14.3523}}	{37.6222, 67.0689}	inf	25.	106.736	{6., 228.}	363.17	0.684394	newly	43.	118.472	{5., 252.}	218.868	0.594594	Reff	1.47606	2.18142	{1.46396, 2.81137}	49.3766	ar	5811.	7604.62	{5620., 9317.}	44.6758
36	R0	{1.61457, 9.93364}	{1.48201, 6.00395}	{{1.25474, 3.26445}, {1.92538, 10.6367}}	{18.4354, 49.1366}	inf	112.	235.75	{49., 459.}	132.277	0.354297	newly	110.	315.475	{69., 518.}	199.614	0.437868	Reff	1.56904	1.41566	{1.10273, 1.89541}	21.9776	ar	6338.	5566.48	{3934., 8253.}	26.4488
37	R0	{1.47831, 7.08139}	{2.33666, 11.7421}	{{1.739, 7.10524}, {2.76203, 15.5722}}	{58.5745, 70.4949}	inf	63.	76.4615	{10., 182.}	84.6642	0.379895	newly	82.	66.5231	{9., 135.}	64.6529	0.431928	Reff	1.45954	2.35344	{1.9191, 2.78151}	61.8478	ar	5530.	8568.82	{7780., 9284.}	55.9841
38	R0	{1.73445, 9.8858}	{1.34087, 3.24111}	{{1.24751, 2.5364}, {1.49267, 4.47101}}	{23.1311, 67.2145}	inf	67.	92.6129	{15., 207.}	88.3004	0.364843	newly	71.	241.645	{31., 491.}	259.882	0.519713	Reff	1.70548	1.3114	{1.16449, 1.42091}	24.3745	ar	6842.	4566.39	{3330., 5912.}	34.5588
39	R0	{1.66621, 11.4824}	{1.55942, 6.73861}	{{1.35758, 4.45823}, {1.92538, 10.6367}}	{14.8132, 43.6031}	inf	179.	278.771	{72., 711.}	90.0878	0.301757	newly	134.	316.371	{55., 575.}	158.102	0.417185	Reff	1.58806	1.50374	{1.25597, 1.83566}	16.7643	ar	6473.	6235.83	{4830., 7808.}	19.5048
40	R0	{1.60694, 9.95741}	{1.25606, 3.53998}	{{1.22544, 3.07157}, {1.28596, 3.98513}}	{21.8351, 64.4488}	inf	141.	140.63	{28., 254.}	54.7938	0.30183	newly	132.	287.222	{54., 486.}	138.412	0.373232	Reff	1.54957	1.10697	{0.974715, 1.26166}	28.563	ar	6625.	3688.	{2975., 4400.}	44.3321
41	R0	{1.55128, 4.71911}	{2.02752, 11.6945}	{{1.2743, 5.03398}, {2.75598, 17.9391}}	{38.7548, 151.71}	inf	6.	2.7	{0., 6.}	81.	0.886148	newly	17.	5.08	{0., 13.}	96.2353	1.50265	Reff	1.54988	2.02659	{1.27417, 2.7546}	38.7911	ar	6141.	3930.2	{1., 9172.}	68.1567
42	R0	{1.60063, 6.81118}	{1.53671, 12.7415}	{{1.21937, 6.0993}, {1.79724, 18.4377}}	{10.5876, 90.0415}	inf	35.	43.6923	{4., 146.}	81.685	0.419918	newly	65.	28.9231	{1., 67.}	70.8876	0.730837	Reff	1.58799	1.59439	{1.29533, 1.82031}	10.4171	ar	6528.	6049.54	{3750., 7416.}	16.8992
43	R0	{1.56986, 5.44652}	{2.09699, 10.546}	{{1.31535, 4.12341}, {2.74056, 16.1449}}	{40.1289, 104.003}	inf	8.	7.0597	{0., 18.}	73.3209	0.541198	newly	23.	9.14925	{0., 27.}	72.9396	0.90669	Reff	1.56751	2.15176	{1.38556, 2.78406}	41.397	ar	6312.	6246.19	{2., 9131.}	46.8979
44	R0	{1.47523, 8.07785}	{1.66946, 8.44172}	{{1.29927, 4.21286}, {2.14401, 13.8218}}	{19.9715, 41.2244}	inf	101.	158.333	{29., 323.}	100.787	0.353404	newly	100.	181.205	{22., 384.}	129.256	0.397813	Reff	1.43968	1.7039	{1.17112, 2.12639}	25.9474	ar	5692.	6736.18	{4317., 8486.}	30.4327
45	R0	{1.6417, 7.69429}	{2.44315, 13.185}	{{1.89254, 8.41615}, {2.96398, 17.7348}}	{49.7841, 74.9178}	inf	45.	79.0571	{12., 169.}	119.492	0.366765	newly	60.	64.0714	{7., 157.}	78.2619	0.404424	Reff	1.62545	2.42844	{1.80738, 2.93671}	50.8281	ar	6735.	8511.49	{7228., 9359.}	31.5491
46	R0	{1.38865, 5.83473}	{1.30396, 3.25671}	{{1.24751, 2.5364}, {1.31535, 3.47086}}	{6.50368, 44.1841}	inf	64.	120.29	{11., 275.}	136.24	0.529944	newly	101.	299.935	{18., 638.}	228.649	0.600951	Reff	1.35977	1.23431	{1.11726, 1.37902}	10.0621	ar	5233.	4243.52	{3598., 5124.}	20.1858
47	R0	{1.45289, 6.87903}	{1.21785, 2.21791}	{{1.19264, 2.01465}, {1.31023, 3.04996}}	{16.1771, 67.7584}	inf	78.	106.429	{2., 188.}	84.6154	0.559668	newly	104.	389.	{40., 692.}	297.115	0.730046	Reff	1.42441	1.15458	{1.04606, 1.34483}	18.9438	ar	5379.	3423.07	{2680., 4848.}	36.3623
48	R0	{1.69416, 9.4831}	{2.21074, 16.3686}	{{1.8484, 12.3777}, {2.49423, 19.4475}}	{30.6332, 72.755}	inf	47.	101.288	{13., 237.}	147.034	0.398152	newly	41.	54.9545	{10., 130.}	85.7724	0.366129	Reff	1.67264	2.19423	{1.83842, 2.46139}	31.2206	ar	6925.	8428.88	{7609., 8934.}	21.7167
49	R0	{1.47843, 7.16286}	{1.31491, 3.40336}	{{1.24751, 2.5364}, {1.31535, 3.47086}}	{11.1499, 52.486}	inf	80.	125.034	{5., 283.}	110.776	0.520725	newly	81.	309.172	{8., 658.}	313.708	0.657317	Reff	1.44664	1.25288	{1.112, 1.35436}	13.8297	ar	5449.	4397.07	{3817., 5356.}	20.6454
50	R0	{1.68843, 5.72427}	{2.0346, 7.08091}	{{1.22544, 2.60601}, {2.79906, 12.6465}}	{33.5074, 53.8701}	inf	14.	4.75	{0., 17.}	82.6429	1.21338	newly	26.	16.64	{0., 67.}	105.154	1.45618	Reff	1.68522	2.03288	{1.22532, 2.78902}	33.579	ar	6800.	3958.81	{1., 9132.}	63.9522
